# The Aryl Hydrocarbon Receptor Mediates the Neurodevelopmental Toxicity of Perfluorooctane Sulfonamide in Zebrafish Larvae

**DOI:** 10.3390/toxics13100832

**Published:** 2025-09-30

**Authors:** Pinyi Chen, Kang Wang, Jie Zhang, Yan Jiang, Tao Chen

**Affiliations:** 1 MOE Education Key Laboratory of Geriatric Diseases and Immunology, The First Affiliated Hospital, Suzhou Medical College of Soochow University, Suzhou 215123, China; chenpy0219@163.com (P.C.); zhangjie_78@suda.edu.cn (J.Z.); 2 Jiangsu Key Laboratory of Preventive and Translational Medicine for Major Chronic Non-Communicable Diseases, Suzhou 215123, China

**Keywords:** PFOSA, AhR, oxidative stress, apoptosis, neurotoxicity

## Abstract

Perfluorooctane sulfonamide (PFOSA), the direct precursor to perfluorooctane sulfonate (PFOS), is widely present in the environment. Research has indicated that PFOSA is cardiotoxic and hepatotoxic, but its impact on neurodevelopment remains unclear. In the current study, we observed that exposure of PFOSA caused neurodevelopmental toxicity in zebrafish embryos in a dose-dependent manner, as evidenced by impaired motor abilities and decreased swimming distance. We then demonstrated that PFOSA exposure downregulated the mRNA expression of neurodevelopment-related genes including a1-tubulin, elavl3, ache and dat. Moreover, PFOSA exposure resulted in dose-dependent oxidative stress, which triggers apoptosis in the brains of zebrafish larvae. We further showed that inhibition of the aryl hydrocarbon receptor (AhR) alleviated the oxidative stress and apoptosis induced by PFOSA, thereby counteracting the neurodevelopmental abnormalities in zebrafish larvae. In conclusion, these findings indicate PFOSA causes neurodevelopmental disorders by inducing oxidative stress and apoptosis through the AhR pathway.

## 1. Introduction

Per- and polyfluoroalkyl substances (PFASs) constitute a class of persistent organic contaminants that have raised significant public health concerns due to their widespread presence in environment and their tendency for bioaccumulation [1]. Perfluorooctane sulfonamide (PFOSA), the immediate precursor of perfluorooctane sulfonic acid (PFOS), is frequently detected in soil, surface water, and groundwater [2]. The concentrations of PFOSA can be up to 15 μg/L in surface waters and 0.09–20,000 μg/kg in surface soil in USA Air Force aqueous film-forming foam-impacted sites [2,3]. Notably, PFOSA has been reported as the only PFOS-related precursor (PreFOS) detected in all water and sediment samples from Taihu Lake, China [3,4]. The primary sources of PFOSA are industrial discharges, the degradation of precursor substances, and domestic wastewater effluents [3,4]. PFOSA was also found to be the most abundant PreFOS in all fish tissues from Taihu Lake [5]. Moreover, the concentrations of PFOSA in tissues of finless porpoises from East China Sea are increasing with time, as observed from the trends between 2009 and 2010 and 2018–2019 [6].

Humans are primarily exposed to PFOSA through the ingestion of contaminated food and water, with concentrations in human blood reaching up to 1.6 μg/L [7]. Importantly, PFOSA can cross the placental barrier, posing potential health risks to developing fetuses [8]. Among 38 tested PFAS compounds, PFOSA was uniquely reported to cause embryonic toxicity in zebrafish at low concentrations [9]. Chen et al. reported that PFOSA, at concentrations ranging from 0.1 to 100 μg/L significantly reduced heartbeat rate, stroke volume, and cardiac output in zebrafish [10]. Additionally, exposure to PFOSA can lead to liver and kidney damage in zebrafish embryos [9,11]. Research on the neurotoxic effects of PFOSA is limited. Slotkin et al. reported that PFOSA has a more detrimental impact on the rat neural cell line PC12 compared to PFOS, perfluorooctanoic acid (PFOA), and perfluorobutane sulfonate (PFBS) [12]. A recent study also demonstrated that PFOSA exposure impaired neurodevelopment in zebrafish embryos in a dose-dependent manner [13]. However, the specific mechanisms behind PFOSA’s neurodevelopmental toxicity remain unclear.

While most PFASs act as agonists of peroxisome proliferator-activated receptors (PPARs), PFOSA predominantly activates the aryl hydrocarbon receptor (AhR) signaling pathway [10,14]. AhR is a ligand-activated transcription factor belonging to the basic helix-loop-helix/Per-ARNT-Sim (bHLH-PAS) family. When no ligands are present, AhR remains inactive in the cytoplasm and is protected from degradation by forming a complex with 90 kDa heat shock proteins (HSP90), the AHR-interacting protein (AIP, also called XAP2), the co-chaperone prostaglandin E synthase 3 (p23), and the protein kinase SRC. Upon ligand binding, AhR translocates to the nucleus, where it heterodimerizes with the aryl hydrocarbon receptor nuclear translocator (ARNT) and binds to xenobiotic response element (XRE) sites in the promoter regions of target genes, thereby regulating their transcription [15]. Recent research shows that AhR plays important roles in multiple cellular processes such as detoxification, cell growth, cell differentiation, immune response, and apoptosis [16]. It has been reported that the AhR signaling pathway mediated 3,6-dibromocarbazole (3,6-DBCZ)-induced neurodevelopmental toxicity in juvenile zebrafish [17]. In addition, maternal exposure to 2,3,7,8-tetrachlorodibenzo-p-dioxin (TCDD) has been found to delay fetal brain growth in rat offspring [18]. The AhR target genes, such as cytochrome P450 CYP1s, can generate reactive oxygen species (ROS) as a byproduct during xenobiotic metabolism [19]. Elevated ROS levels may induce oxidative damage, impair neural structure and function, and lead to neuronal injury or death [20].

Apoptosis, also known as programmed cell death, can be classified into two types: intrinsic apoptosis, which is mediated by mitochondria, and extrinsic apoptosis, which is mediated by death receptors. It is widely acknowledged that apoptosis is crucial for neurodevelopment, particularly in shaping the developing brain [21]. However, excessive apoptosis is often a significant factor in developmental neurotoxicity [22]. Numerous environmental chemicals with neurodevelopmental toxicity, such as metals, pesticides, and endocrine-disrupting compounds, can trigger neuronal cell apoptosis [22]. Our recent findings indicate that exposure to PFOSA leads to apoptosis through AhR-mediated oxidative stress in the hearts of zebrafish embryos [14]. Consequently, we propose that PFOSA induces apoptosis and disrupts neurodevelopment through the AhR/ROS axis.

The zebrafish (*Danio rerio*) model offers distinct advantages for assessing developmental neurotoxicity, including genetic tractability, optical transparency, and conserved neurotransmitter systems [23]. Zebrafish behavioral phenotypes have emerged as sensitive biomarkers for neurotoxicant screening, with locomotor deficits strongly correlating with neurodevelopmental outcomes in mammals [23,24]. In this study, we characterize PFOSA-induced neurodevelopmental toxicity in zebrafish embryos and explore the role of the AhR/ROS axis in this process.

## 2. Materials and Methods

### 2.1. Chemicals

PFOSA (CAS: 754-91-6, purity 94.9%) was obtained from Scrbio, China. The AhR inhibitor CH223191 (CH, CAS:301326-22-7, purity 99.64%) and the ROS scavenger *N*-Acetylcysteine (NAC, CAS: 616-91-1, purity > 98%) were purchased from AbMole, Shanghai China and Beyotime, Shanghai, China, respectively. All these chemicals were dissolved in dimethyl sulfoxide (DMSO) to obtain a stock solution and stored in −80 °C.

### 2.2. Zebrafish Husbandry and Chemical Exposure

Fertilized eggs of wild-type AB strain zebrafish were collected and put into dishes with E3 medium consisting of 5 mM NaCl, 0.17 mM KCl, 0.33 mM CaCl_2_, and 0.33 mM MgSO_4_, and were kept at 28.5 °C under a 14:10 h light: dark cycle. At 2 h post-fertilization (hpf), healthy embryos were randomly distributed into six-well glass plates, with 80 embryos per well. PFOSA was dissolved in DMSO at 50 mg/L to prepare a stock solution, and serial dilutions (1:2) were made before each experiment. Embryos were treated with PFOSA at different concentrations (6.25, 12.5, 25, 50 μg/L) in the presence or absence of CH (0.05 μM) or NAC (0.25 µM) until 72 hpf. DMSO (0.01%, *v*/*v*) served as the vehicle control. Exposure solutions were refreshed every 24 h.

### 2.3. Real-Time Reverse Transcription-Polymerase Chain Reaction (RT-PCR)

Total RNA was isolated from the head of zebrafish larvae using Trizol reagent (Vazyme, Nanjing, China). RNA purity and concentration were evaluated by using a NanoDrop 2000 spectrophotometer (NanoDrop Technology, Wilmington, DE, USA). complementary DNA (cDNA) was synthesized using a commercial reverse transcription kit (Vazyme, Nanjing, China). RT-PCR amplifications were conducted on an ABI 7500 real-time-PCR system (Applied Biosystems, Foster City, CA, USA) using SYBR Green PCR Master Mix (Vazyme, Nanjing, China). Primer sequences are listed in Table 1. The thermal cycling conditions included an initial denaturation for 2 min at 50 °C, followed by 10 min at 95 °C, then 40 cycles of 95 °C for 15 s and annealing at 60 °C for 60 s. Relative gene expression levels were normalized to β-Actin and calculated using the 2^−△△CT^ method.

### 2.4. Zebrafish Behavioral Tests

Locomotion activity was assessed as reported previously [25]. Briefly, at 72 hpf, healthy zebrafish embryos exhibiting normal development were selected under an optical microscope and individually transferred to wells of a 48-well plate filled with 1 mL of E3 medium. The locomotor assay began with a 15 min habituation period under constant light, followed by a 20 min light/dark transition stimulus. This transition phase was designed with two full cycles of alternating light conditions, each cycle consisting of 5 min of darkness and 5 min of light. The locomotor behavior was analyzed using Danio Vision observation system (Noldus Information Technology, Wageningen, The Netherlands), and parameters such as cumulative movement duration, total swimming distance, and mean velocity were measured and analyzed with EthoVision XT 15 software (Noldus, The Netherlands). Twelve embryos were tested per exposure group and all trials were independently replicated in triplicate.

### 2.5. Reactive Oxygen Species (ROS) Detection

To evaluate oxidative stress status, ROS levels were assessed using DCFH-DA (2′,7′-Dichlorodihydrofluorescein diacetate) staining. Ten embryos at 72 hpf from each group were incubated with 5 μg/mL DCFH-DA in darkness for 30 min. After incubation, unincorporated dye was removed by washing the embryos three times with phosphate-buffered saline (PBS, 1×). Representative fluorescence images of the embryos were acquired using a fluorescence microscope (SMZ18, Nikon, Tokyo, Japan) under identical exposure settings. This experiment was conducted in triplicate.

### 2.6. Detection of Apoptosis

Acridine orange (AO) staining was utilized to assess apoptosis levels in the brain region of zebrafish larvae. Briefly, ten zebrafish embryos from each group were immersed in acridine orange solution (5 mg/L) in darkness for 30 min. After being rinsed three times with PBS to remove excess dye, the larvae were anesthetized in tricaine (MS-222). Fluorescence images of the brain area were immediately captured using a stereo fluorescence microscope (SMZ18, Nikon). The fluorescence intensity, indicative of apoptotic cell density, was quantified using ImageJ software (version 1.8.0). To avoid potential confounding effects on neurological development, phenylthiourea (PTU) was not used to maintain larval transparency. To ensure the reliability of the apoptotic signals, background subtraction was applied to predefined regions during image analysis. Future studies using the skin-transparent Casper model would enable more accurate apoptosis quantification.

### 2.7. Statistical Analysis

All experiments were replicated at least three times under independent conditions. Statistical significance was performed using one-way ANOVA followed by Dunnett’s or Tukey’s multiple comparison tests, as appropriate. Data are presented as mean ± SEM (Standard Error of the Mean). A *p* values <0.05 was deemed statistically significant.

## 3. Results

### 3.1. PFOSA Exposure Induces Locomotor Deficits and Behavioral Preference Alterations and in Zebrafish Larvae

**Figure 1:**
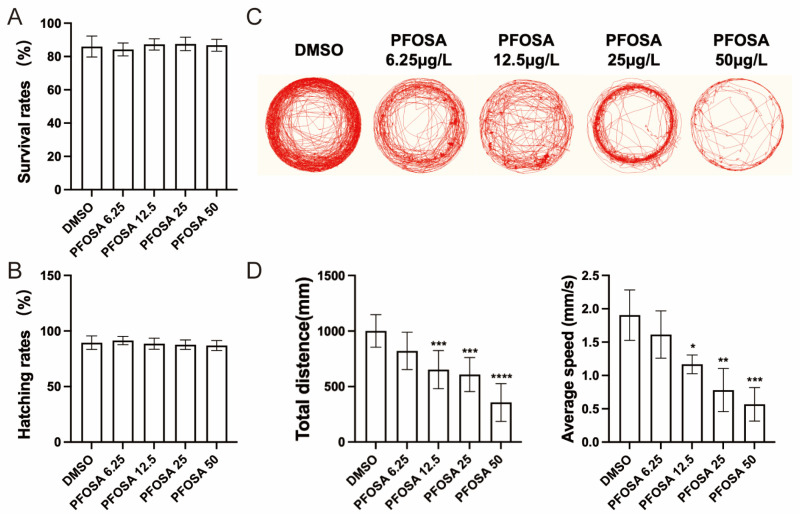
Effects of the motor behavior of zebrafish larvae exposed to PFOSA. (**A**) Survival rates. (**B**) Hatching rates. (**C**) Representative locomotor traces in two light-dark cycles in larval zebrafish. (**D**) Total distance and average speed in two light-dark cycles. PFOSA 6.25, 12.5, 25, 50: PFOSA at different concentrations (μg/L). * *p* < 0.05, ** *p* < 0.01, *** *p* < 0.001, **** *p* < 0.0001.

As shown in Figure 1A,B, no significant differences in survival rates or hatching rates were observed in zebrafish embryos exposed to PFOSA at different concentrations (0–50 μg/L) when compared to DMSO controls. However, behavioral analysis revealed that PFOSA exposure led to a dose-dependent reduction in the complexity of movement trajectories (Figure 1C). Moreover, exposure to PFOSA significantly reduced total swimming distance and decreased swimming speed at concentrations exceeding 6.25 µg/L (Figure 1D).

### 3.2. PFOSA Exposure Impairs Neuronal Differentiation

**Figure 2:**
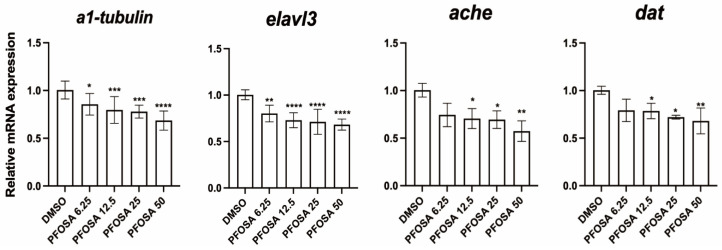
Effect of zebrafish embryo exposed to PFOSA on neurodevelopment-related genes. * *p* < 0.05, ** *p* < 0.01, *** *p* < 0.001, **** *p* < 0.0001.

As illustrated in Figure 2, PFOSA exposure downregulated the mRNA expression levels of α1-tubulin (an early neuronal differentiation marker) and elavl3 (a marker of mature neurons) in a dose dependent manner. Notably, PFOSA even at the lowest tested concentration of 6.25 μg/L significantly decreased the transcriptional levels of these two gene. Additionally, PFOSA at concentrations above 6.25 μg/L significantly decreased the mRNA levels of ache and dat (acetylcholinesterase and dopamine transporter, both are key neurotransmitter system components).

### 3.3. PFOSA Exposure Leads to Oxidative Stress and Apoptosis in Zebrafish Brains

**Figure 3:**
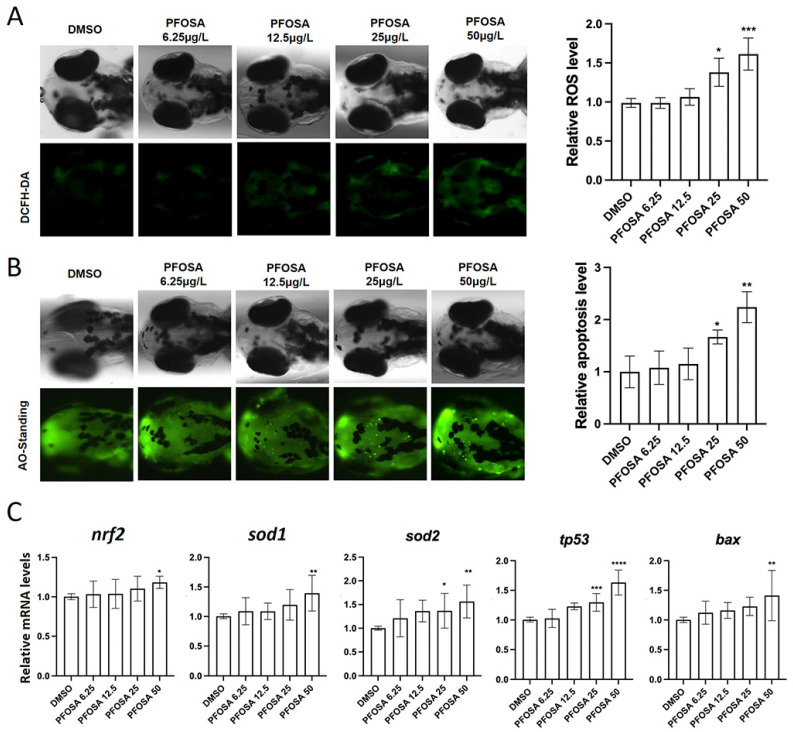
PFOSA causes oxidative stress and apoptosis in the brain of zebrafish embryos. (**A**) Intracellular ROS levels. (**B**) Apoptosis detected by AO staining. (**C**) The expression levels of nrf2, sod1, *sod2, tp53* and *bax* in zebrafish embryos from different treatment groups. All data are expressed as mean ± standard deviation. *, significant difference compared to the control group (* *p* < 0.05, ** *p* < 0.01, *** *p* < 0.001, **** *p* < 0.0001).

PFOSA at dose levels above 12.5 induced a concentration-dependent increase in ROS production in embryonic heads (Figure 3A). We further demonstrated that PFOSA induced apoptosis in a concentration-dependent manner, evidenced by increased apoptotic bodies in the head of zebrafish larvae (Figure 3B). In consistent, the mRNA expression levels of the oxidative stress-related gene sod2 and the pro-apoptotic gene tp53 were elevated in the groups exposed to PFOSA at 25 and 50 µg/L (Figure 3C). The mRNA levels of nrf2a, sod1 and bax were also significantly increased in the group with the highest concentration of PFOSA (Figure 3C).

### 3.4. PFOSA Triggers Apoptosis via AhR-Mediated Oxidative Stress

**Figure 4:**
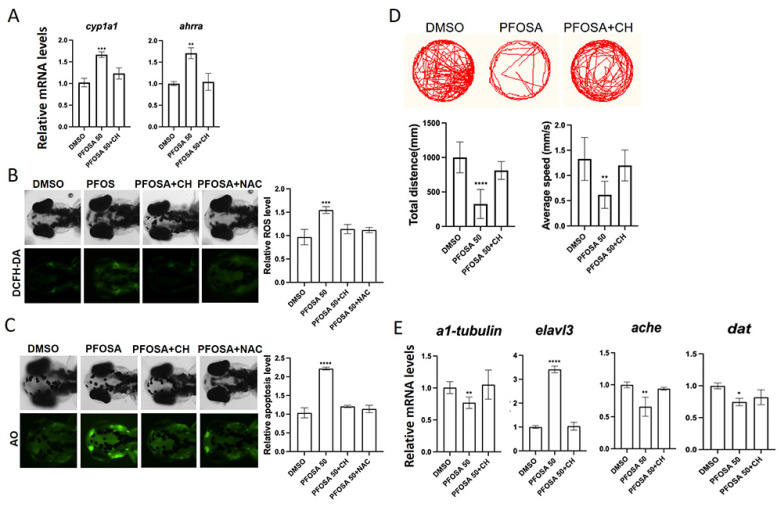
The AhR/ROS axis mediates PFOSA-induced apoptosis and neurodevelopmental toxicity in zebrafish embryos. (**A**) Relative mRNA levels of AhR downstream genes. (**B**) Intracellular ROS levels. (**C**) Apoptosis detected by AO staining. (**D**) Representative locomotor traces, total distance, and average speed. (**E**) Relative mRNA levels of genes involved in neurodevelopment. (* *p* < 0.05, ** *p* < 0.01, *** *p* < 0.001, **** *p* < 0.0001).

We first observed that the mRNA levels of cyp1a1 and ahrra, two prototypical AhR downstream genes, were elevated in high concentration (50 µg/L) PFOSA samples but returned to normal levels in the presence of CH, indicating that AhR signaling was activated by PFOSA (Figure 4A). We then demonstrated that the addition of the AhR inhibitor CH as well as the ROS scavenger NAC counteracted oxidative stress and apoptosis in the brain region of zebrafish larvae exposed to PFOSA (Figure 4B,C). Notably, co-treatment with CH abolished the behavioral deficits in locomotor activity caused by PFOSA (Figure 4D). The aberrant expression patterns of neuro-differentiation genes (*α1-tubulin, elavl3, ache* and *dat*) in PFOSA samples were also returned to control levels in the PFOSA plus CH group (Figure 4E).

## 4. Discussion

Behavioral analysis is recognized as an effective approach for evaluating neurotoxicity [26]. In this study, we observed that zebrafish embryos’ exposure to PFOSA resulted in a dose-dependent reduction in the complexity of movement trajectories, total swimming distance, and swimming speed. Moreover, the mRNA expression levels of neurodevelopmental related genes—including *α1-tubulin*, *elavl3*, *ache* and *dat* —were downregulated in the head of zebrafish embryos exposed to PFOSA. These findings are in line with a recent study that reported that PFOSA exposure reduced the locomotor activity of larval fish and decreased the expression levels of elavl3 and ache [13]. α1-tubulin is an essential component of the microtubule cytoskeleton and plays a key role in the structural and functional integrity of axons and dendrites [27]. Elavl, a neural-specific RNA-binding protein, is involved in neurogenesis [28]. AChE activity is crucial for the inactivation of acetylcholine at nerve terminals and for the proper functioning of the sensory and neuromuscular systems [29]. Dat as a dopamine transporter regulates synaptic dopamine levels, which are vital for social behavior in fish [30]. The downregulation of these genes suggests that PFOSA exposure not only impacts neurogenesis but also disrupted the structure and function of nervous system.

Neural development relies on tightly regulation of neurogenesis and apoptosis [31]. In this study, we observed that PFOSA exposure induced a dose-dependent increase in apoptotic bodies in the heads of zebrafish larvae. Additionally, the mRNA levels of pro-apoptotic genes including *tp53* and *bax* were elevated following PFOSA exposure. The transcription factor p53, encoded by *tp53*, plays a key role in cellular stress responses. Activated p53 can elicit apoptosis in various cell types including neurons [32]. Bax, a member of the Bcl-2 family, is a direct target of p53. Activation of Bax by p53 can cause mitochondria permeabilization and initiate intrinsic apoptosis [33]. The increase in apoptotic bodies and the upregulation of *tp53* and *bax* have been reported in the hearts of zebrafish embryos exposed to PFOSA, suggesting that PFOSA might trigger apoptosis through a similar mechanism across different organs [14].

Oxidative stress arises when there is an imbalance between the generation of ROS and the body’s capability to neutralize them, often resulting in cellular damage and death [34]. It has been established that oxidative stress, as a main driver of apoptosis, is a critical factor in neurodevelopmental toxicity [35]. We recently reported that PFOSA exposure induced oxidative stress and apoptosis in zebrafish embryonic hearts [14]. In this study, we observed a dose-dependent elevation in ROS production and the upregulation of oxidative related genes, including *nrf2*, *sod1*, and *sod2*, in the head of zebrafish larvae exposed to PFOSA. NAC is among the most widely used antioxidant agents [36]. The addition of ROS scavenger NAC not only mitigated oxidative stress but also diminished apoptosis in the brain area of zebrafish larvae exposed to PFOSA. Nrf2 serves as a the key transcription factor in the antioxidant defense system, while Sod1 and Sod2 are crucial for detoxifying superoxide radicals [37]. Our findings suggest that antioxidant treatment could be a promising therapeutic strategy to alleviate PFOSA-induced damage during neural development.

AhR, which is expressed in the vertebrate brain in the early developmental stages, plays an essential role in neurogenesis [38,39]. Overactivation of AhR has been shown to promote neuronal cell apoptosis in the hippocampus of mice [40]. Recent studies showed that AhR is involved in PFOSA-caused cardiac defects in zebrafish embryos [10,14]. Here, we demonstrated that PFOSA activated AhR in the head of zebrafish larvae, evidenced by the overexpression of downstream genes *cyp1a1* and *ahrra*. Cyp1a1, a prototypical AhR target gene, is important not only for xenobiotic metabolism, but also plays a key role in AhR-induced oxidative stress [41]. We have previously reported that AhR activation by benzo[a]pyrene leads to ROS overproduction through Cyp1a1 in zebrafish [42]. There are two Ahrr isoforms in zebrafish, with Ahrra primarily responsible for regulating AHR signaling during development [43]. In consistent, we observed that inhibition of AhR diminished oxidative stress, apoptosis and behavior abnormalities caused by PFOSA exposure.

## 5. Conclusions

In summary, our results indicate that PFOSA exposure elicits oxidative stress and promotes apoptosis in the brains of zebrafish embryos via AhR activation, leading to abnormal neurodevelopment. Our study contributes to understanding the molecular mechanisms underlying the neurodevelopmental toxicity of PFOSA, highlighting the ecological and health risks of this persistent pollutant. As this research focuses on early development, additional studies are needed to investigate the neurotoxic impacts of PFOSA on later developmental phases or adult fish to assess the reversibility of these effects. Moreover, further research using mammalian models will be valuable in assessing the potential risks of PFOSA to human health.

## Figures and Tables

**Table 1:** Primer sequences.

Genes	GenBank No.	Forward (5′–3′)
*β-actin*	NM_131031.2	CGAGCAGGAGATGGGAACC CAACGGAAACGCTCATTGC
*elavl3*	NM_131449.1	TGGTCTGCAGTTTGAGACCGTTGA
*α1-tubulin*	NM_194388.3	AATCACCAATGCTTGCTTCGAGCC TTCACGTCTTTGGGTACCACGTCA
*dat*	NM_131755.1	AGACATCTGGGAAGGTGGTG ACCTGAGCATCATACAGGCG
*ache*	NM_131846.3	CCCTCCAGTGGGTACAAGAA GGGCCTCATCAAAGGTAACA
*nrf2*	NM_182889.1	TCGGGTTTGTCCCTAGATG AGGTTTGGAGTGTCCGCTA
*sod1*	NM_131294.1	CCGGACTATGTTAAGGCCATCT ACACTCGGTTGCTCTCTTTTCTCT
*sod2*	NM_199976.1	GTCGTCTGGCTTGTGGAGTG TGTCAGCGGGCTAGTGCTT
*tp53*	NM_001271820.1	CCCGGCGATCATGGATTTAG CCACATGCTCGGACTTCTTATAG
*bax*	NM_131562.2	GGCTATTTCAACCAGGGTTCC TGCGAATCACCAATGCTGT
*cyp1a1*	NM_131879.2	GCATTACGATACGTTCGATAAGGAC GCTCCGAATAGGTCATTGACGAT
*ahrra*	NM_001035265.2	GCGCATCAAGAGCTTCTGCAGCGTGTT CCACTGACGACCAGCGCAAACCCT

## Data Availability

Data will be made available on request.
